# Stage 2 Registered Report: How subtle linguistic cues prevent unethical behaviors

**DOI:** 10.12688/f1000research.25573.2

**Published:** 2020-11-16

**Authors:** Wen Guo, Huanxu Liu, Jingwen Yang, Yuqi Mo, Can Zhong, Yuki Yamada

**Affiliations:** 1 Graduate School of Human-Environment Studies, Kyushu University, Fukuoka, Japan; 2 Faculty of Arts and Science, Kyushu University, Fukuoka, Japan

**Keywords:** cheating, persuasion, attention, moral, self construal, labeling

## Abstract

**Background:** Differences in descriptions can influence people’s evaluations and behaviors. A previous study by Bryan and colleagues suggested that subtle linguistic differences in ethical reminders can differentially prevent readers’ unethical behavior. The present study tried to replicate the previous finding in the Japanese context (Experiment 1); additionally, we explored the influence of unfamiliar Japanese instruction words that captured participants’ attention (Experiment 2).

**Methods:** In two online experiments, participants were asked to make 10 coin-tosses and report the number of “heads” results, which would indicate the amount of money that they could earn. In Experiment 1, we analyzed the difference in the number of “heads” results as reported by 768 participants under three conditions with different instructions (“Don’t cheat” vs. “Don’t be a cheater” vs. baseline as a control). In Experiment 2, we conducted an extended experiment with an additional task in which more attention was directed toward the text.

**Results:** In Experiment 1, we successfully replicated the results of the original experiment. The results of Experiment 2 showed no evidence that the results in Experiment 1 were influenced by attentional factors.

**Conclusions:** In conclusion, the results of the present study supported the hypothesis that self-identity-related words of moral reminder curb unethical behaviors more effectively.

Stage 1 report: https://doi.org/10.12688/f1000research.20183.4

## Introduction

When people behave dishonestly, they usually downplay the seriousness of the dishonest act (e.g., Monin & Jordan, 2009; Steele, 1988), weakening the link between the dishonesty and one’s self-identity (e.g., Bandura, 1999) to avoid the correspondent inference ( Jones & Nisbett, 1972; Ross, 1977) that one is the kind of person who behaves dishonestly. According to self-concept maintenance theory, individuals in general strive to create and maintain an image of themselves as good and ethical people ( Markus & Wurf, 1987; Mazar *et al.,* 2008).

According to Blasi (1984), a moral person is one for whom moral categories and moral notions are central, essential, and important to self-understanding. Morals cut deeply to the core of what and who such people are as individuals. However, one study revealed that highly constructed self-identities are associated with more unethical behaviors ( Cojuharenco *et al.,* 2012).

Regarding ethical behavior, a moral-character model has been proposed, where moral character consists of motivation, ability, and identity elements ( Cohen & Morse, 2014). Moral identity here refers to being disposed toward valuing morality and wanting to view oneself as a moral person. This disposition should be considered when attempting to understand why people who behave unethically tend to apply a variety of strategies to weaken the behavior–identity link ( Bandura, 1999). The use of “euphemistic labeling” to describe one’s attributes and weaken the link regarding language should also be included in this disposition.

Different ways of description can easily influence people’s evaluation and judgment about something, even if they have a wealth of previously established knowledge ( Fausey & Boroditsky, 2010). For instance, using a transitive verb (agentive description, e.g., “Timberlake ripped the costume”) to describe an accident makes participants significantly more likely to blame the actor compared to the same description with the words changed to an intransitive verb (nonagentive description, e.g., “The costume ripped”). Another study found that, for children aged 5–7 years old, when a noun label was employed to describe a character (e.g., “She is a carrot-eater”) rather than a verbal predicate (e.g., “She eats carrots whenever she can”), their judgment about those characteristics would be more stable over time ( Gelman & Heyman, 1999). The same phenomenon has been demonstrated regarding self-perception ( Walton & Banaji, 2004). It is possible that language has some effect in this category ( Gelman *et al.,* 2000) because when nouns are used to refer to something, one may have a deeper understanding of it, which is noted to “enable inductive inferences” ( Gelman & O’Reilly, 1988).

Once the subtle description is used to refer to oneself, a noun label may have a stronger effect. Bryan *et al.* (2011) found that more people would choose to vote if they heard the words “be a voter” rather than “to vote” on the day before election day. Additionally, research showed that, compared to “helping,” “being a helper” encouraged more children to conduct kind behaviors toward others ( Bryan *et al.,* 2014). However, subsequent research found that although “being a helper” can lead to more kind behaviors initially, once there is a setback, the backlash may also be stronger accordingly ( Foster-Hanson *et al.,* 2020). The reason underlying this phenomenon is as follows: as category labels, nouns bear a strong link to identity and may lead to self-doubt once one fails.

According to Bryan *et al.* (2011), the effect of noun expression comes from a motivation-driven process. When a noun is involved with a positive identity such as “voter” and “helper,” people simply see themselves as voters or helpers and they produce more correlated behaviors; When the noun is involved with undesirable (negative) identities, however, these kind of words should cause people to avoid correlated behaviors. Highlighting a self-identity word will prevent unethical behaviors to some degree ( Bryan *et al.*, 2013).

In social psychology, experiments of priming of unethical behaviors and its subsequent prevention typically involve money or time ( Gino & Mogilner, 2014; Gino & Pierce, 2009; Mogilner & Asker, 2009; Vohs *et al.,* 2006). A mere exposure to money is associated with unethical outcomes ( Kouchaki *et al.,* 2013). In Gino *et al.*’s experiment (2014), participants were asked to complete a scrambled-sentences task using some money-related words or time-related words; results showed that priming time (rather than money) makes people behave more ethically.

In contrast, another experiment by Bryan *et al.* (2013) allowed experimenters to prevent unethical behaviors through semantic priming. They manipulated the task’s instructions by changing the use of verbs (“Don’t cheat”) to noun labels (“Don’t be a cheater”) to inhibit participants from engaging in unethical behaviors. The self-identity related group (“don’t be a cheater”) had significantly lower proportion of unethical cheating behaviors.

In the present study, we aim to replicate Experiment 3 of Bryan *et al.* (2013), for the following reasons:

First, the participants in Experiment 1 in Bryan *et al.* (2013) were asked to think of a number from 1 to 10. If the number was even, they were paid $5; if it was odd, there was no reward. Bryan *et al.* (2013) paid for even numbers because it has been reported that participants typically show a strong bias toward odd numbers in a random number generation task ( Kubovy & Psotka, 1976), but this oddness bias had not been confirmed for betting behaviors. Furthermore, an even or odd number participants think of is just imaginary, occurring in one’s inside world, not an external real event; hence, it is difficult to use it as an index of falsification. An index used for cheating should emphasize that participants’ reports can differ from the fact. Thus, we abandoned the method of Bryan *et al.* (2013) Experiment 1. In their Experiments 2 and 3, they used a coin-tossing task: participants were asked to toss a coin and receive a reward corresponding to the result of their coin flips. We choose this method for our experiment because tossing a coin induces a real external event, which is more objective and operable, and hence it is better than thinking of a number to measure cheating behavior. In addition, compared with Experiment 2 in the original study, which just used two conditions, “cheater” and “cheating”, a baseline group was included in Experiment 3, which made Experiment 3 more complete in its design—an approach we followed also.

Moreover, we found that the effect size in Experiment 3 was small ( *f* = 0.302 in G*Power (significance level α = 0.05, power level 1-β = 0.95), meaning that Experiment 3 required at least 174 participants; in fact, only 99 people joined the original research. From this, we suppose that the effect size in Experiment 3 was overvalued.

According to the above review, high levels of self-identity and the willingness of individuals to maintain a positive self-view should prevent unethical behaviors. We predict that the self-relevant noun “cheater” will curb cheating behaviors more significantly than the verb “cheating” and the baseline condition (in which there is no reminder in the instruction).

## Methods

### Experiment 1

Our experiment was conducted online in a private and impersonal way, which means that participants did not meet or be expected to meet the experimenters. We aimed to replicate Experiment 3 of Bryan *et al.* (2013), in which there were three conditions: “cheater,” “cheating,” and “baseline”; in the baseline condition, a reminder about cheating was not mentioned.

***Participants.*** Participants were users of the Yahoo! Crowdsourcing Service in Japan. Participants were required to meet the *a priori* criterion that they were native Japanese speakers. We conducted a pilot test to determine the shortest time in which one could reasonably participate in the experiment in good faith. This pilot test is detailed in a later section ( *Outlier extraction*). Participants were excluded if they completed the experiment faster than the pilot test time. Repeat participation was prevented.

***Procedure.*** At the beginning of the experiment, demographic information of participants’ age and gender was collected. Online instructions indicated that a recent controversial article has claimed to report the first scientific evidence for paranormal phenomena ( Bem, 2011); this was the same cover story used in the original study.

We asked the participants to find a coin at home and, while trying to influence the outcome of each toss with their minds, flip the coin 10 times, making it land on “heads” as often as possible. They were asked to ensure that they were “properly motivated.” For every toss landing on “heads,” they received 10 T-points (≈ 10 yen) as a reward. The instructions indicated that participants would receive a reward corresponding to the result of their coin flips. Noting that the average Yahoo! Crowdsourcing user receives two T-points for one task, the reward we set was five times that amount. Even those participants who reported a chance level of five “heads” received 25 times more points than for a normal task. This reward should be large enough for some participants to engage in cheating behavior.

Participants were randomly assigned the three conditions. All three conditions were posted on the crowdsourcing website, and a person who participates in one of the conditions cannot participate in the others. This resulted in passive randomization. In the “cheater” and “cheating” conditions, the manipulation was embedded in the instructions. We referred to the instructions in the original experiment ( Bryan *et al.,* 2013) and translated it into Japanese, as follows:

NOTE: Please don’t [cheat/be a cheater] and report that one or more of your coin flips landed heads when it really landed tails! Even a small [amount of cheating/number of cheaters] would undermine the study, making it appear that psychokinesis is real.

[嘘をつかない/嘘つきにならない]でください。コイントスの結果，実際にはコインの裏が出たのに表が出たと報告しないでください。

たとえわずかでも[嘘があると/嘘つきがいると]，この研究を台無しにし，念力が実在するように見せてしまいます。

The manipulation was also embedded in the instructions on the next page, where participants log the outcomes of their 10 coin-flips. At the top of the page, a message was displayed in large red words: “嘘をつかないでください/嘘つきにならないでください” This means “PLEASE DON’T [CHEAT/BE A CHEATER]” as in the original experiment ( Bryan *et al.,* 2013).

In the baseline condition, the instructions were the same as above, except that the cheating message was not mentioned.

***Power analysis and sample size.*** Because Experiment 3 of Bryan *et al.* (2013) did not report the effect size, η ^2^, first, we calculated the effect size of the analysis of variance (ANOVA) result from the *F* and *df* values. Bryan *et al.* (2013) reported the statistics of their one-way ANOVA as *F*(2, 96) = 4.38, *p* = .015. Hence, we calculated η ^2^ based on Cohen’s (1973) method, as η ^2^ = .0836. Then, we calculated the effect size, *f*, as follows: *f* = √(η ^2^/(1 – η ^2^) = 0.302. The small sample size may overestimate the effect size so, as a replication convention (e.g., Nitta *et al.,* 2018), we halved the effect size of the original experiment, and used G*Power 3.1.9.3 ( Faul *et al.,* 2009) to conduct a power analysis (i.e., to 0.151). In G*Power, we set the significance level α = 0.05, power level 1-β = 0.95, and effect size *f* = 0.151. According to the conditions of the original experiment, we divided the participants into three groups. The required total sample size was 681, with 227 participants in each group; therefore, we tried to recruit at least 681 participants, and data collection did not exceed 810 participants. This stopping rule was set because it was difficult for us to limit the number of participants to exactly 681, due to the characteristics of the simultaneous participatory online recruitment system; therefore, we allowed for up to 120% of the required sample size (i.e., 810). If more than 810 people participated in the experiment, we selected the data of the first 810 participants based on the time stamp and used this for the analysis. Also, we set the number of participants (max. 365 males and 445 females) to match the gender distribution of the original study (male: female = .45:.55).

***Exclusion criteria*.** For our online experiment, we established a minimum completion time (MCT) for inclusion in the final sample by asking five colleagues who were unfamiliar with this experiment to complete the experiments as fast as possible, then calculating the mean completion time. Specifically, each colleague performed a coin toss ten times; after each toss they recorded the result on the experiment website. This pilot test did not include the attempt to motivate psychokinesis and measured only the required time of the coin toss and recording. Bryan *et al.* (2013) also used the MCT as an extraction criterion. We excluded those participants who completed it faster than the MCT, because they might have rushed through the experiment and failed to complete it in good faith.

***Data analyses.*** In this study, the dependent variable was the mean number of “heads” reported. In the original experiment, a one-way ANOVA and *t*-test were performed. Specifically, the ANOVA was performed for analyzing the main effect of the three groups. A problem in the original study was that the authors did not report adjustments for any significance level in subsequent multiple comparisons. Therefore, in the present study, we used a one-way ANOVA and Tukey’s method for the multiple comparisons. Additionally, in order to check the cheating in each group, the original study performed one-sample *t*-tests between the mean number of “heads” reported and the chance level (i.e., 50%; 5 times out of 10 flips). These analyses were performed using jamovi (version 1.0.5). The original results are summarized in Table 1.

**Table 1.** Results of Experiment 3 of Bryan *et al.* (2013).

Analysis types		Reported *p*-value	Degree of freedom	Effect size
Main effect: three groups		.015	96	*f* = 0.302
*t*-test	“cheating” vs “cheater”	.013	96	*d* = 0.71
	“cheater” vs baseline	.004	96	*d* = 0.66
	“cheating” vs baseline	> .80	96	*d* = 0.05
	“cheating” vs “chance”	< .0005	36	*d* = 0.79
	baseline vs “chance”	< .0005	35	*d* = 0.78
	“cheater” vs “chance”	> .30	25	*d* = 0.19

Moreover, as the dependent variable was based on the counts of “heads” reported and that the 10 coin tosses were nested within each participant, a quasi-Poisson or Poisson regression was used for exploratory analyses. In the (quasi-)Poisson model, the variance was assumed to be the mean multiplied by a dispersion parameter ( Ma *et al.,* 2014). Dispersion parameters with a value greater than one indicate that overdispersion exists; in this case, quasi-Poisson regression will be performed. Thus, which analysis to used depends on the result of variance and the mean of “heads” counts. We first tested the original hypothesis. Then, information of gender and age were added as predictors to establish a regression model.

### Experiment 2

This experiment was employed as an extended, conceptual replication of Experiment 3 in the original study ( Bryan *et al.,* 2013). Our Experiment 2 was only performed when the results of Experiment 1 successfully replicated those of the original experiment. In the original experiment, the numbers of heads claimed in the “cheater” condition was significantly lower than that in the “cheating” and baseline conditions, but no difference was found between the “cheating” and baseline conditions. Here we cannot easily interpret the non-significant results based on self-identity alone. We aimed to test whether lower levels of attention to the instruction in the “cheating” condition reduced the effectiveness of preventing dishonest behaviors in our Experiment 1. Thus, we conducted Experiment 2, adding a “cheating” with attention task condition in which we used tasks concerning an instruction to ensure that participants’ attention was captured (e.g., Folk *et al.,* 1992; Folk *et al.,* 2002). When we translated the instruction into Japanese, we felt the unfamiliarity of a “cheater” condition in a Japanese language situation. Participants in our experiment might find that the reminder “don’t be a cheater” commands extra attention because of this sense of deviation. Therefore, even if the result of the original experiment was completely reproduced in our Experiment 1, it would not fully support the finding of the original experiment, as the reason for the possible different dishonest behavior rates between the “cheating” and “cheater” conditions in our Experiment 1 might be that the participants in the “cheating” group paid relatively less attention to the instruction; for this reason, “cheating” might have worked weakly as a moral reminder in this condition. Because the experiments are conducted online, it was difficult to ensure that the participants have actually seen and understood the instruction; in addition, it was also possible that the participants ignored the instructions of Experiment 1 due to satisficing, (e.g., Chandler *et al.,* 2014; Oppenheimer *et al.,* 2009; Sasaki & Yamada, 2019), further diminishing the effect of the unattended reminder (i.e., “cheating”). In this Experiment 2 we addressed these attention-related effects.

Noticeably, the main difference between our Experiment 1 and the original Experiment 3 lay in the different language used in the instruction. Thus, if our Experiment 1 was a successful replication, we would then choose to focus on the expression used in the Japanese instruction, rather than the English instruction of the original Experiment 3.

To support this approach, we conducted a preliminary experiment, asking participants to evaluate their familiarity with certain expressions in Japanese. The expressions “Don’t cheat” and “Don’t be a cheater” were translated into Japanese, and native speakers evaluated their familiarity with them (1: not familiar to 5: very familiar) via an Internet survey on Yahoo! Crowdsourcing. The protocol of this experiment was registered on the Open Science Framework ( Guo *et al.,* 2020). The results showed that the familiarity rating score in the “cheater” condition was significantly lower than that in the “cheating” condition, *t*(64) = 6.73, *p* < .001, Cohen’s *d* = 0.834. Hence, we conjectured that the anticipated difference in the results between the “cheating” and “cheater” conditions in Experiment 1 may partly occur due to differences in attention paid to the instruction, instead of the preservation of a positive self-image proposed by the original study ( Bryan *et al.,* 2013). This meant that part of the effect of the “cheater” condition was due to the unfamiliar expression, which attracts people’s attention then played a role in preventing them from conducting unethical behavior. See *Extended data* for details about this experiment.

In our Experiment 2, we manipulated the way in which participants saw the instructions to explore the differences between the “cheating” and baseline conditions. Experiment 2 comprised three conditions: “cheating,” “cheating” with attention task, and baseline. We predicted that the “cheating” with attention task condition would be more effective in curbing unethical behaviors than the “cheating” and baseline conditions, because the task would arouse more attention. While the instruction in the “cheating” condition was in large red capital letters, this should entail no significant difference compared with baseline.

***Procedure.*** The procedure for Experiment 2 was identical to that of Experiment 1, except for important differences in two aspects. In Experiment 2, we focused on whether the participants read the instructions as diligently as we expect. First, we omitted the original “cheater” condition and included instead another “cheating” condition (i.e., “cheating” with attention task condition). Second, in the “cheating” with attention task condition, we added a task page in which participants were asked to choose the exact expression (i.e., “Don’t cheat”) that appeared on the screen from three sample sentences. We reminded participants of this task in advance to ensure they read the instructions carefully.

***Power analysis and participants.*** Because the power analysis of Experiment 2 was the same as in Experiment 1, we intended to recruit participants in the same way as Experiment 1. The minimum completion time was also established for participants to be included in the final sample. This exclusion standard was similar to that in Experiment 1.

***Data analyses.*** In Experiment 2, the dependent variable was the mean number of “heads” reported. We still used a one-way ANOVA and Tukey’s method for the multiple comparisons. To check the cheating rate in each group, a one-sample *t*-test between the mean number of “heads” reported and the chance level (50%) was analyzed. The data of participants who failed to provide the right answer to the attention task were not used for further analysis. Another analysis by a (quasi-)Poisson regression model was also performed to explore the contribution factors of cheating counts.

## Study timeline

After Stage 1 acceptance, our colleagues were asked to complete the pilot test to calculate the MCT. Then, we posted our experiments on the Yahoo! Crowdsourcing Service to recruit participants. The experiments and subsequent analysis were conducted from March 29, 2020 to July 10, 2020.

### Ethical approval and consent to participate

The present study received approval from the psychological research ethics committee of the Faculty of Human-Environment Studies at Kyushu University (approval number: 2019-004). Completion of experiments by participants was regarded as consent to participate; they also had the right to withdraw from the experiment at any time without providing a reason. In addition, we protected participants’ personal information. Because this study was conducted online, even if participants engaged in cheating behaviors, we could not identify them or meet the participants face-to-face.

## Results

### Experiment 1

We recruited 1298 users of the Yahoo! Crowdsourcing service who clicked on a survey called “Coin Game.” Of these participants, 803 met our minimum completion time (MCT) criterion for good faith during the course of the experiment. Moreover, we deleted the data of participants exceeding the limit we set to match the gender distribution of the original study; finally, we used data from 768 participants (males = 315, females = 445, no response = 8, *M _age_* = 45.68) for analyses.

**Figure 1.**
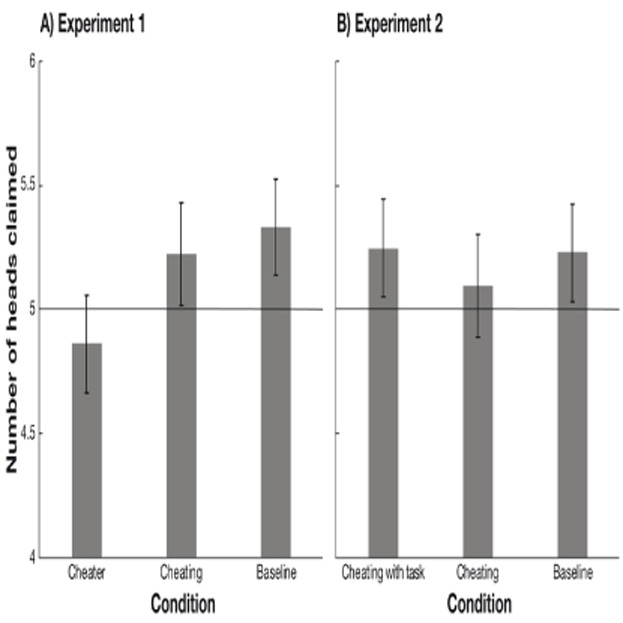
The mean number of “heads” in each instruction condition in Experiment 1 (Panel **A**) and Experiment 2 (Panel **B**). Error bars indicate 95% confidence intervals of the mean.

In this study, the dependent variable was the mean number of “heads” reported. We performed a one-way ANOVA and Tukey’s method for the post hoc comparisons. The results of ANOVA showed a significant main effect of condition ( *F* (2,765) = 5.86, *p* = .003, *f* = 0.123). Multiple comparisons showed that participants in the “cheater” condition reported having obtained significantly lesser “heads” than the participants in the “cheating” condition ( *t* (765) = 2.554, *p* = .029, Cohen’s *d* = 0.22) and the baseline condition ( *t* (765) = 3.24, *p* = .004, Cohen’s *d* = 0.29). The “heads” reported in the baseline condition had no significant difference with the “cheating” condition ( *t* (765) = 0.754, *p* = .731, Cohen’s *d* = 0.07). The results of Experiment 1 suggested that participants instructed with “cheater” had a significantly lesser cheating rate than those instructed with “cheating” and those that were uninstructed ( Figure 1A). Hence, Experiment 1 successfully replicated the results in the original study ( Bryan *et al.*, 2013).

Moreover, we compared each condition to a chance level (i.e., five times) to check bias in each group. The “cheating” ( *M* = 5.22) and baseline ( *M* = 5.33) groups had a significantly higher rate than five ( *t* (265) = 2.20, *p* = .029, Cohen’s *d* = 0.135 and *t* (241) = 3.31, *p* = .001, Cohen’s *d* = 0.212, respectively). The number of “heads” in the “cheater” ( *M* = 4.86) group did not significantly differ from five ( *t* (259) = 1.36, *p* = 0.176, Cohen’s *d* = 0.084). In this respect too, we succeeded in replicating the results of the original experiment.

The dependent variable was based on counts of reported “heads” and 10 trials were nested within each participant. A Poisson regression analysis on the number of reported “heads” was performed. We first added “condition” as a factor into the model. The results indicated that condition ( *χ ^2^* (2) = 6.23, *p* = 0.044) significantly contributed to explaining variance related to the number of “heads.” In contrast, when participants’ gender and age were used as predictors for the regression model, the results indicated that gender ( *χ ^2^* (2) = 1.11, *p* = .575) and age ( *χ2* (58) = 26.40, *p* = 1.00) did not significantly predict the number of “heads.”

Although we successfully replicated the results in the original study, it was still premature to conclude that the subtle difference in the instruction affected the cheating behavior. In particular, as there is a significantly different degree of familiarity between the words “cheater” and “cheating” in the Japanese instruction (see our preliminary experiment), there was still a possibility that attentional factors might have influenced the cheating. Therefore, we conducted Experiment 2.

### Experiment 2

We recruited 1255 users of the Yahoo! Crowdsourcing service. Among these, 800 (males = 365, females = 425, *M _age_* = 43.35) met the MCT criterion for good-faith participation and were used for analyses. The number of participants by gender did not exceed the maximum sample size we had set.

In Experiment 2, three conditions, “cheating,” “cheating” with attention task, and baseline groups, respectively, were employed. The analysis was identical to Experiment 1. The result of the one-way ANOVA did not show a significant main effect of condition [ *F* (2, 797) = 0.667, *p* = .514, *f =* 0.045 ( Figure 1B)]. The cheating behavior in the “cheating” with attention task condition did not significantly decrease more than the baseline and “cheating” conditions ( *t* (797) = 0.957, *p* = .604, Cohen’s *d* = 0.01 and *t* (797) = -1.034, *p* = .555, Cohen’s *d* = 0.09).

Moreover, we also performed a *t*-test between each condition and a chance level of five. The “cheating” with attention task condition ( *M* = 5.25) and baseline ( *M* = 5.23) condition had a significantly larger number than five ( *t* (242) = 2.380, *p* = .018, Cohen’s *d* = 0.153 and *t* (286) = 2.389, *p* = .018, Cohen’s *d* = 0.141, respectively). The number of “heads” in the “cheating” condition had no significant difference with five ( *t* (269) = 0.937, *p* = .350, Cohen’s *d* = 0.057).

A Poisson regression analysis on the number of reported “heads” was also performed. Condition [ *χ ^2^* (2) = 0.628, *p* = .731] did not significantly contribute to explain variance related to the number of “heads.” Additionally, gender [ *χ ^2^* (2) = 0.728, *p* = .695] and age [ *χ ^2^* (57) = 27.139, *p* = 1.00] did not significantly predict the number of “heads.”

These findings do not suggest that the settings made for participants to pay full attention to the reminder instruction significantly reduced the incidences of cheating. Therefore, it is reasonable to assume that the significant effect of “cheater” in Experiment 1 was not due to excessive attention to the instruction.

## Discussion

In this study, we directly replicated Experiment 3 of Bryan *et al.* (2013) (Experiment 1) and addressed potential attentional effects related to translation (Experiment 2) using a Japanese sample. These registered experiments were perfectly performed without any deviation from the protocol. As a result, in Experiment 1, we successfully replicated the original results of Bryan *et al.* (2013). We found a significant main effect of condition similar to the original study, and the “Don’t be a cheater” had a minimal number of reported “heads” ( *M*=4.86), which meant participants in this group had the lowest rate of dishonest behavior. However, we could not conclude that the participants in the “cheater” group have lesser cheating behaviors than those in the “cheating” group. As we predicted in the Stage 1 protocol and confirmed in a preliminary experiment, the unfamiliar expression of “Don’t be a cheater” in the Japanese context would have made participants pay more attention to this sentence; hence, it would have made for a more effective reminder. In other words, “Don’t cheat” in the Japanese context might be ignored by some participants and lose its role as a reminder. Therefore, there were more cheating behaviors in the “cheating” condition.

To examine the attentional factor on cheating behaviors, we added an additional “Don’t cheat” condition with a recognition task for the reminder. We aimed to test whether the lower levels of attention caused by a familiar instruction (“Don’t cheat”) in the “cheating” condition reduced the effectiveness of preventing unethical behavior in our Experiment 1. One of the mechanisms of the theory of self-concept maintenance relies on the attention to standards of one’s conduct ( Mazar *et al.*, 2008). As Mazar *et al.* (2008) found, when reminded of their standards, people had to face their actions, and therefore, could be more honest. However, in Experiment 2, no significant main effect of conditions was shown, which clarified that the proper attention to the reminder “Don’t cheat” was still ineffectual in curbing the cheating behavior. We did not obtain results consistent with Mazar *et al.* (2008). Thus, we excluded the role of the attentional factor in verb-based and noun-based reminders in the Japanese context. The results supported the hypothesis proposed by Bryan *et al.* (2013) that, in such cases, undesired self-relevant nouns can cause people to avoid a particular behavior and such nouns influence behavior by emphasizing its implications for identity ( Bryan *et al.*, 2011).

Although we successfully replicated the original study, some differences were present in terms of results. Note that the effect size of the present study was considerably smaller than the original study ( *f* = 0.302 for the original study and *f* = 0.123 for the present study). Similarly, in the multiple comparisons, as per the trend (“cheating” – “cheater” ** = 0.71 and “cheater” – baseline = 0.66 for the original study vs. “cheating” – “cheater” = 0.22 and “cheater” – baseline = 0.29 for present study), it is likely that there was some inflation of effect size due to the small sample size in previous studies (99 participants in the original study vs. 768 participants in the present study). Another replication of the original study by Savir & Gamliel (2019) also obtained results with a lower effect size. They added positive-valence appeals to check the effectiveness of self-appeals in reducing unethical behavior. In their meta-analysis, “cheater” was actually more effective than “cheating”, whereas they reported a lower effect size (overall estimate of 0.17) as compared with Bryan *et al.* (2013) (overall estimate of 0.80). In the present study, the effect size was comparable to the previous replication study ( Savir & Gamliel, 2019).

Besides the comparison between conditions, to check bias in each group, we compared the “heads” times in each condition in both experiments to a chance level. Inconsistent with the original experiment and Experiment 1, in Experiment 2, there was no significant difference between the “cheating” condition and the chance level. This inconsistency in the results between the present experiments does not affect the interpretation of the main hypothesis we tried to test here. Yet, whether this “cheating” reminder does or does not affect behavior is inconsistent across studies as well, and hence it will be interesting to further investigate what factors define the difference. Note that the present study showed that the effect size of the reminder itself is very small, so it is clear that no promising hypothesis exists at this point as a crud factor ( Orben & Lakens, 2020).

The present study contributes to research across cultures and to the generalization of the original effect. Previously published studies employing a similar method have studied this effect in US and Israel ( Bryan *et al.*, 2013; Savir & Gamliel, 2019), but to the best of our knowledge, the subtle linguistic effects of moral reminders on cheating have not been investigated in Asia. The results of this study also provide a cross-cultural validity of the inhibitory effects of different lexical-based warnings on unethical behaviors.

In the present study, we selected a sample of Japanese people in Asia (instead of Americans in the original experiments) and set up an authentic online survey environment as in the original study (instead of a laboratory experiment) to ensure that participants behave naturally. Given that in the two language contexts (Japanese and American English) self-identity words curb unethical behaviors more effectively, we expect that our results with users of a crowdsourcing platform can be generalized to other online contexts. However, given that the priming of unethical behavior in the present study (the same as Bryan *et al.*, 2013) was based on the coin flip paradigm, the pattern of results, which might hold only for the instant coin flip paradigm, do not generalize to curbing all kinds of unethical behaviors. It should also be noted that linguistic expressions conveying the same meaning may still vary greatly in two different language families, depending on the cultural habits and the language structure itself (as in our preliminary experiment, “Don’t be a cheater” was not a familiar expression in Japanese, as it is not usually used in that way); therefore, it is still necessary to consider the generality of this effect in countries with a similar linguistic system in future research. Considering the results of the Poisson regression analysis, we have no reason to believe that the results depend on other characteristics of the participants. It is unclear at this time whether other materials and contextual factors could be constraints on generality ( Simons *et al.*, 2017).

A limitation that we want to discuss is the considerably high rate of dropout: 495 people of 1298 (38.1%) and 455 people of 1255 (36.2%) in the two experiments, respectively. In future online surveys, we may need to recruit participants over 150% of the required sample size to ensure an adequate number of participants. There were advantages and disadvantages to conducting the cheating experiment online; because of anonymity, participants were free from social expectations and could cheat more easily ( Grym & Veronica, 2016). However, we did not have a true grasp of whether participants completed the experiment in good faith (e.g., Sasaki & Yamada, 2019).

## Conclusion

In conclusion, this study successfully replicated the results of Experiment 3 of Bryan *et al.* (2013) as a registered report without any deviation from the protocol, and excluded the possibility of the attentional factor when the reminder was translated to Japanese. We closely followed their experimental design and analytic methods. The results of our study supported the hypothesis that self-identity-related nouns of moral reminder curbed unethical behaviors more effectively than when the same meaning was expressed using verbs; furthermore, they supported the cross-linguistic validity of these findings. Moreover, we found that the attentional factor showed no effect in decreasing unethical behaviors in the Japanese context. Future research should also consider personal traits when focusing on the inhibiting effects of language on individual unethical behaviors.

## Data availability

### Underlying data

Open Science Framework: How subtle linguistic cues prevent unethical behaviors, https://doi.org/10.17605/OSF.IO/AXDHV ( Guo *et al.,* 2020).

This project contains the following underlying data:

- Data collected for Experiment 1 (XLSX format, uploaded 15/07/2020)
- Explanation for dataset of Experiment 1 (RTF format, uploaded 13/07/2020)
- Data collected for Experiment 2 (XLSX format, uploaded 13/07/2020)
- Explanation for dataset of Experiment 2 (RTF format, uploaded 13/07/2020)

### Extended data

Open Science Framework: How subtle linguistic cues prevent unethical behaviors, https://doi.org/10.17605/OSF.IO/AXDHV ( Guo *et al.,* 2020).

This project contains the following extended data:

- Protocol for the pilot study conducted for Experiment 2 (PDF format, uploaded 31/07/2019)
- Data collected for the pilot study conducted for Experiment 2 (XLSX format, uploaded 23/07/2019)

Data are available under the terms of the Creative Commons Attribution 4.0 International license (CC-BY 4.0).
