# Supplementary material for: Stage 2 Registered Report: How subtle linguistic cues prevent unethical behaviors
Source: F1000Res. 2020 Nov 16;9:996. Originally published 2020 Aug 18. [Version 2] doi: 10.12688/f1000research.25573.2 (PMC7590892; doi:10.12688/f1000research.25573.2)
Supplement: Peer review history

# Peer review: Reviewer response for version 1

**DOI:** 10.5256/f1000research.28223.r69709

**Published:** 2020-10-26

**Authors:** Michiko Asano

**Affiliations:** 1 Department of Psychology, College of Contemporary Psychology, Rikkyo University, Saitama, Japan

This study nicely replicates Experiment 3 of Bryan *et al. *(2013), which showed that a self-relevant noun of moral reminder (i.e., “Please don’t be a cheater”) curbed unethical behaviours more effectively than a verb (i.e., “Please don’t cheat”) did, in an online survey environment with Japanese speakers. The results of Experiment 2 of the current study, which addressed potential attentional effects related to translation, strengthened their claim.

 I only have minor suggestions, as described below, and approve this manuscript for publication.

**Minor suggestions:**

1. The last sentence of the second paragraph of Introduction “However, one study revealed that highly constructed self-identities are associated with more unethical behaviors (Cojuharenco *et al.*, 2012).”:
  It is not very clear to me what point the authors are trying to make by citing this study here.
2. The fourth paragraph of Discussion: “Inconsistent with the original experiment and Experiment 1, in Experiment 2, there was no significant difference between the “cheating” condition and the chance level. This discrepancy is not important because it is not relevant to the claimed hypothesis... Note that the present study showed that the effect size of the reminder itself is very small, so it is clear that no promising hypothesis exists at this point as a crud factor (Orben & Lakens, 2020).”:
  I understand that the lack of a significant difference between the “cheating” condition and the chance level is not relevant to the hypothesis of Experiment 2, and the effect size of the reminder itself is small. However, I still feel the authors’ claim here (“This discrepancy is not important”) is too strong and perhaps needs to be toned down because it is the prerequisite for this line of study that cheating behaviours occur in the “cheating” condition.

Is the work clearly and accurately presented and does it cite the current literature?

Yes

If applicable, is the statistical analysis and its interpretation appropriate?

Yes

Are the data able to test the authors’ proposed hypotheses by satisfying the approved outcome-neutral conditions?

Yes

Did the authors adhere precisely to the registered experimental procedures? If not, has an explanation been provided regarding any change?

Yes

Are all the source data underlying the results available to ensure full reproducibility?

Yes

Is the study design appropriate and is the work technically sound?

Yes

Are the conclusions drawn adequately supported by the results?

Yes

Are the introduction, rationale and stated hypotheses the same as the approved Stage 1 submission?

Yes

Are any unregistered post hoc analyses added by the authors justified, methodologically sound and informative?

Not applicable

Are sufficient details of methods and analysis provided to allow replication by others?

Yes

Reviewer Expertise:

NA

I confirm that I have read this submission and believe that I have an appropriate level of expertise to confirm that it is of an acceptable scientific standard.

# Response

**Published:** 2020-10-31

**Authors:** Yuki Yamada

**Affiliations:** Kyushu University, Japan

Thank you for reviewing our registered report again! We have responded to each of your comments as follows.

**Minor suggestions:**

*1. The last sentence of the second paragraph of Introduction “However, one study revealed that highly constructed self-identities are associated with more unethical behaviors (Cojuharenco et al., 2012).”:*

*It is not very clear to me what point the authors are trying to make by citing this study here.*

**Reply:** In Cojuharenco et al. (2012), high levels of self-identities construction elicited more unethical behaviors. This finding was inconsistent with the conclusion of the experiment in Bryan et al. (2013) that we intended to replicate, thus here we cited this research with different conclusions in order to place more emphasis on the importance of replicating the results of Bryan's experiment. However, to avoid excessive and unnecessary changes of the introduction (the stage 1 manuscript), we decided only to explain it here, but do not add extra explanations to the manuscript.

*2. The fourth paragraph of Discussion: “Inconsistent with the original experiment and Experiment 1, in Experiment 2, there was no significant difference between the “cheating” condition and the chance level. This discrepancy is not important because it is not relevant to the claimed hypothesis... Note that the present study showed that the effect size of the reminder itself is very small, so it is clear that no promising hypothesis exists at this point as a crud factor (Orben & Lakens, 2020).”:*

*I understand that the lack of a significant difference between the “cheating” condition and the chance level is not relevant to the hypothesis of Experiment 2, and the effect size of the reminder itself is small. However, I still feel the authors’ claim here (“This discrepancy is not important”) is too strong and perhaps needs to be toned down because it is the prerequisite for this line of study that cheating behaviours occur in the “cheating” condition.*

**Reply:** Thank you for your constructive suggestion. As you pointed out, we have to admit that we discussed the “cheating” condition in Experiment 2 with a too strong tone. It is meaningful to explore the effectiveness of “don’t cheat” reminder in different circumstances, thus here we cannot assert that this discrepancy is not important. We toned down the discussion and modified the corresponding part of the manuscript as follows:

 This inconsistency in the results between the present experiments does not affect the interpretation of the main hypothesis we tried to test here. Yet, whether this “cheating” reminder does or does not affect behavior is inconsistent across studies as well, and hence it will be interesting to further investigate what factors define the difference.

# Peer review: Reviewer response for version 1

**DOI:** 10.5256/f1000research.28223.r69708

**Published:** 2020-09-22

**Authors:** Sergio Cervera-Torres

**Affiliations:** 1 Leibniz-Institute für Wissensmedien (IWM), Tübingen, Germany; 2 Salumedia Labs SLU, Sevilla, Spain

The study nicely replicates, in a Japanese sample, the findings by Bryan *et al*. (2013) about how subtle linguistic cues prevent unethical behaviour based on a coin-tossing task. 

 As a reviewer of Stage 1 of this study, I must say that all my major concerns have been successfully amended. I only have minor suggestions:

 - **Introduction**: I still think that the storyline could be improved a bit. I find unclear whether the statement "In general, we believe that highlighting a self-identity word..." at the beginning of the second paragraph is an especial belief of the authors or an argument. Maybe it could suit better at the end of the paragraph "According to Bryan et al. (2011)...".

 - **Experiment 1**. I would use Inclusion (or exclusion) criteria as a heading instead of "outlier extraction" and move the section before the "data analyses" section. 

 - **Experiment 2**. You use three different expressions to name the new condition: "cheating" with task condition, "cheating with attention task" condition, and "don't cheat" condition with a recognition task. I suggest being consistent through the paper and use, "cheating with attention task". In the procedure, I would use different wording for "..we deleted the original "cheater condition and added..." such as "...we omitted the original cheater condition and included instead..."

 - **Discussion**. In the second paragraph, you state "we aimed to test whether the effect of the reminder was enhanced by participants' excessive attention to the unfamiliar expression [cheater] in the Japanese context". This is not exactly what you did.

 As you state in the introduction of Experiment 2  "We aimed to test whether lower levels of attention to the instruction in the “cheating” condition reduced the effectiveness of preventing dishonest behaviours in our Experiment 1".

 The difference is subtle but important.

Is the work clearly and accurately presented and does it cite the current literature?

Partly

If applicable, is the statistical analysis and its interpretation appropriate?

Yes

Are the data able to test the authors’ proposed hypotheses by satisfying the approved outcome-neutral conditions?

Yes

Did the authors adhere precisely to the registered experimental procedures? If not, has an explanation been provided regarding any change?

Yes

Are all the source data underlying the results available to ensure full reproducibility?

Yes

Is the study design appropriate and is the work technically sound?

Yes

Are the conclusions drawn adequately supported by the results?

Yes

Are the introduction, rationale and stated hypotheses the same as the approved Stage 1 submission?

Yes

Are any unregistered post hoc analyses added by the authors justified, methodologically sound and informative?

Not applicable

Are sufficient details of methods and analysis provided to allow replication by others?

Yes

Reviewer Expertise:

Cognitive & e-Health Psychology.

I confirm that I have read this submission and believe that I have an appropriate level of expertise to confirm that it is of an acceptable scientific standard.

# Response

**Published:** 2020-10-31

**Authors:** Yuki Yamada

**Affiliations:** Kyushu University, Japan

Thank you for reviewing our registered report again! We have responded to each of your comments as follows.

*1.- **Introduction**: I still think that the storyline could be improved a bit. I find unclear whether the statement "In general, we believe that highlighting a self-identity word..." at the beginning of the second paragraph is an especial belief of the authors or an argument. Maybe it could suit*

*better at the end of the paragraph "According to Bryan et al. (2011)...".*

**Reply:** In the second paragraph, we wanted to state how the self-identity influences ethical behaviors through the moral core of a person. However, this topic was also discussed in Bryan et al. (2013) and it makes sense to put it at the end of the sixth paragraph of the introduction as your suggestion. As this is a revision of a part of the manuscript of stage 1, please let us add a footnote before the “Data Availability” part to explain this modification does not change the logic or the hypothesis ****that we registered in our stage 1 manuscript.

*2.- **Experiment 1**. I would use Inclusion (or exclusion) criteria as a heading instead of "outlier extraction" and move the section before the "data analyses" section.*

**Reply:** Thank you for your suggestion. We have replaced the “Outlier extraction” with “Exclusion criteria” and set the “Exclusion criteria” section before the “Data analyses” section.

*3.- **Experiment 2**. You use three different expressions to name the new condition: "cheating" with task condition, "cheating with attention task" condition, and "don't cheat" condition with a recognition task. I suggest being consistent through the paper and use, "cheating with attention task". In the procedure, I would use different wording for "..we deleted the original "cheater condition and added..." such as "...we omitted the original cheater condition and included instead..."*

**Reply: **According to your suggestion, the expression of the new condition has been unified as “cheating” with attention task. Besides, we also modified the corresponding part in the manuscript.

*4.- **Discussion**. In the second paragraph, you state "we aimed to test whether the effect of the reminder was enhanced by participants' excessive attention to the unfamiliar expression [cheater] in the Japanese context". This is not exactly what you did.*

*As you state in the introduction of Experiment 2 "We aimed to test whether lower levels of attention to the instruction in the “cheating” condition reduced the effectiveness of preventing dishonest behaviors in our Experiment 1". The difference is subtle but important.*

**Reply**: The purpose of Experiment 2 was to explore the role of the attention factor on unethical behavior in different samples from Experiment 1. As you said, we should revise the expression to describe our work more clearly, so we have rephrased the description of the discussion as follows:

 We aimed to test whether the lower levels of attention caused by a familiar instruction (“Don’t cheat”) in the “cheating” condition reduced the effectiveness of preventing unethical behavior in our Experiment 1.
